# Evaluating the therapeutic potential of BSA-reduced mussel-derived selenium nanoparticles to mitigate copper sulfate-induced hepatic damage and neurodegeneration in a zebrafish model

**DOI:** 10.3389/fgene.2025.1522370

**Published:** 2025-05-19

**Authors:** Suganiya Umapathy, Ieshita Pan

**Affiliations:** Department of Medical Biotechnology, Institute of Biotechnology, Saveetha School of Engineering, Saveetha Institute of Medical and Technical Sciences, Chennai, Tamil Nadu, India

**Keywords:** liver fibrosis, reactive oxygen species, selenium nanoparticles, antioxidant, zebrafish

## Abstract

**Introduction:**

Liver fibrosis is the abnormal accumulation of extracellular matrix and eventual formation of fibrous scar in response to chronic liver injury, which can be triggered by increased levels of reactive oxygen species. The brain-liver axis is a crucial communication pathway that significantly influences the intricate interactions between hepatic function and brain health. Selenium, as a source of selenoproteins, plays a vital role in antioxidant defense systems. The extraction of selenium from mussels leverages their natural bioaccumulation, providing a biocompatible source. Selenium nanoparticles are known for their potential antioxidant activity and can be employed to regulate ROS levels to overcome hepatic damage.

**Methods:**

Selenium nanoparticles were synthesized from mussel-extracted selenium and stabilized with bovine serum albumin. The zebrafish models exposed to copper sulfate were treated with selenium nanoparticles (5-25 μg/ml). This study evaluated their potential role as antioxidants against hepatic damage induced by copper sulfate *in vivo* in the zebrafish model.

**Results:**

The bovine serum albumin stabilized selenium nanoparticles reduced for 30 minutes and 1 hour were spherical with a size of 19 and 16 nm. Stabilized selenium nanoparticles reduced for 30 minutes (25 μg/ml) showed significant *in vitro* reactive oxygen species scavenging activity and improved *in vivo* antioxidant enzyme levels by decreasing lipid peroxidation and nitric oxide levels. Histopathological examination revealed a delay in the progression of copper sulfate-induced hepatic damage, and upregulated the expression of antioxidants, while the hepatic and mitochondrial damage markers were downregulated.

**Conclusion:**

In conclusion, bovine serum albumin-reduced selenium nanoparticles can be a promising therapeutic antioxidant for protecting against reactive oxygen species-induced hepatic damage and neurodegeneration.

## Highlights

• Green-synthesized and stabilized nano-selenium is a potential therapeutic agent for controlling Cu-induced ROS, which can lead to neurodegeneration.
• In zebrafish, nano-Se ultimately reduces Cu-induced hepatic damage by maintaining the integrity of liver cells.
• 25 μg/mL Se-NPs are potential *in vitro* ROS scavengers. They increase SOD, CAT, GSH, and GPX levels while reducing the levels of LPO and NO in treated zebrafish.
• Se-NPs show promise as neuroprotective agents for restoring AChE levels and cognitive functions in zebrafish

## 1 Introduction

Liver fibrosis is a pathological condition characterized by excessive accumulation of extracellular matrix proteins, including collagen, that results from chronic liver injury. It represents a wound-healing response to liver damage, where the normal liver tissue is replaced by scar tissue (Bataller and Brenner, 2005; Addissouky et al., 2024). This fibrotic process occurs in response to various factors such as viral hepatitis (hepatitis B and C), chronic alcohol abuse, non-alcoholic fatty liver disease, and autoimmune diseases (Athuluri-Divakar and Hoshida, 2019). Recent studies suggest that liver health extends beyond its functions, influencing the brain through the brain–liver axis. This communication network is crucial for maintaining metabolic balance (Vegas-Suárez et al., 2022; De Cól et al., 2024). The liver is a vital organ with a remarkable capacity to regenerate (Qian et al., 2022). However, when repeated or persistent injury occurs, the balance between tissue repair and destruction is disrupted (Schilrreff and Alexiev, 2022). This triggers the activation of hepatic stellate cells, the main cell type responsible for fibrogenesis, leading to the deposition of fibrous scar tissue (Chen et al., 2024). Over time, the accumulation of scar tissue impairs liver function and architecture (Luangmonkong et al., 2018). A key factor in its development is the overproduction of reactive oxygen species (ROS). Exposure to copper sulfate (CuSO_4_) is known to induce liver fibrosis by generating excessive ROS and causing oxidative stress (Seffner et al., 1997; Wang et al., 2016). Oxidative stress, caused by an imbalance between the production of ROS and the body’s ability to detoxify these reactive molecules, is a major contributing factor to various diseases, including liver fibrosis and neurodegenerative disorders (Chen et al., 2020).

With growing interest in sustainable and biocompatible nanomaterials, research is increasingly focused on optimizing nanoparticle synthesis, improving biocompatibility, and understanding their interactions within biological systems (Ranjha et al., 2022). Nanoparticles can be synthesized through diverse methods, such as environmentally friendly green synthesis and physical and conventional chemical techniques (Ijaz et al., 2020; Osman et al., 2024). This versatility enables innovative solutions in fields such as medicine, environmental remediation, energy, and materials science, contributing to advancements (Sen et al., 2024). Among the various nanomaterials, selenium nanoparticles (Se-NPs) have gained significant attention due to their distinctive characteristics and potential applications in multiple fields, particularly in biomedicine (Karthik et al., 2024; Umapathy et al., 2024).

Selenium is an essential micronutrient that is crucial in maintaining cellular health, primarily due to its involvement in the body’s antioxidant defense systems (Bjørklund et al., 2022). It is a key component of selenoproteins, which are enzymes that help neutralize the effects of harmful ROS and protect cells from damage caused by oxidative stress (Jomova et al., 2023). By reducing ROS, selenium helps protect cells from damage and prevent the progression of these conditions (Zhang F. et al., 2023). Mussels are particularly valuable as a natural source of selenium due to their ability of bioaccumulating this element from their environment. Through a process known as bioaccumulation, mussels concentrate selenium in their tissues, making them a rich and sustainable source of this vital micronutrient (Kristan et al., 2015; Abderrahmani et al., 2021; Dey et al., 2024).

However, when selenium is reduced to its nanoparticle form, its surface area increases, allowing for more efficient interaction with ROS and other reactive molecules. This nano-formulation significantly enhances the antioxidant capacity of Se-NPs to mitigate oxidative stress by scavenging ROS more effectively than selenium alone (Zhao et al., 2018; Bisht et al., 2022). This ability to modulate biological pathways and enhance the immune response has opened new avenues for treating conditions such as neurodegenerative diseases, liver disorders, and cancer (Khurana et al., 2019; Chen et al., 2022). This study focuses on exploring the therapeutic potential of Se-NPs reduced with bovine serum albumin (BSA) for their efficacy in protecting against hepatic damage and neurodegeneration caused by exposure to CuSO_4_ in zebrafish.

## 2 Materials and methods

### 2.1 Extraction of selenium from mussels

#### 2.1.1 Collection and preparation of mussel

Mussels (domain: Eukaryota; kingdom: Animalia; phylum: Mollusca; class: Bivalvia; order: Mytilida; family: Mytilidae; genus: *Perna*; species: *viridis*) were freshly collected whole from Kasimedu Fishing Harbor, Tondiarpet (N 13° 7′22.4292″, E 80° 17′36.4272″), Chennai. Sand and other detritus were thoroughly cleaned out of the mussels. After cracking open the shells, the tissue was carefully scraped and cleaned with distilled water. Excess water in the collected tissue was removed and dried overnight. The dried mussel tissue was then stored at 4°C for use in further experiments.

#### 2.1.2 Extraction of selenium

##### 2.1.2.1 Extraction with 0.8% saline

A saline solution (0.8%) was prepared by dissolving 8 g of sodium chloride (NaCl) in 1,000 mL of distilled water. Using a mortar and pestle, 8.5 g of mussel tissue was ground along with 100 mL of the 0.8% saline solution. The mixture was centrifuged at room temperature (37°C) for 30 min at 5,000 rpm until no particulates were visible. The collected pellet was dried in a hot-air oven at 60°C and then pulverized with a mortar and pestle (Kristan et al., 2015).

##### 2.1.2.2 Extraction with 50 mM Tris–HCl buffer pH 7.4

Fresh Tris–HCl buffer (pH 7.4) was prepared by dissolving 4.44 g of HCl and 2.65 g of Tris base in 1,000 mL of distilled water. An amount of 8.5 g of the mussel tissue was mashed with 100 mL of the Tris–HCl buffer using a mortar and pestle. Fine selenium powder was obtained following the outlined procedure for selenium purification (Kristan et al., 2015).

### 2.2 Synthesis of Se-NPs

Se-NPs were synthesized by reducing and stabilizing mussel-derived selenium using BSA. In summary, 0.2 g of mussel-derived selenium was dissolved in 50 mL of distilled water using a sonicator. In a round-bottom flask, 50 mL of the dissolved mussel-derived selenium was mixed with 25 mL of 1% BSA. The process was carried out at two different intervals of 30 min and 1 h, both without and with a stabilizer (5% BSA) using a heating mantle. The mixture was centrifuged at 5,000 rpm for 30 min at 37°C. The collected pellets were dried at 60°C in a hot-air oven. Once completely dried, the pellets were ground into a fine powder using a mortar and pestle (Nie et al., 2016; Salem et al., 2022). These synthesized Se-NPs were utilized for further experiments.

### 2.3 Characterization of mussel-extracted selenium and Se-NPs

The Se-NPs were extensively analyzed using various analytical methods. PerkinElmer Optima 5300 DV Inductively Coupled Plasma Optical Emission Spectroscopy (ICP-OES) was utilized to determine trace element concentrations (Machat et al., 2002). A Bruker D8 Advance Powder XRD was employed for X-ray diffraction (XRD) and crystallographic investigations within a range of 10 and 80 degrees of 2θ (Ramamurthy et al., 2013). The presence of functional groups was examined using Fourier-transform infrared (FTIR) spectroscopy. Thermo Fisher Scientific’s Nicolet Summit FTIR instrument, operated in the diffuse reflectance mode, was used for this analysis. A total of 16 scans covering a wavenumber range of 400 to 4,000 cm^−1^ were obtained using the DTGS KBr detectors (Ramamurthy et al., 2013). The obtained spectra were plotted with the wavenumber (cm^−1^) on the X-axis and transmittance (%) on the Y-axis using OriginPro 8.5 Software. Zeta potential was determined using the nanoPartica SZ-100V2 (Horiba) (Ramamurthy et al., 2013). To analyze the surface morphology of the Se-NPs, a Hitachi Model S-3400 N scanning electron microscope (SEM) was used. The SEM was operated with semiconductor secondary electron (SE) detection in the high vacuum mode at 15 kV. The obtained images were further analyzed for size distribution using ImageJ software (Ramamurthy et al., 2013).

### 2.4 *In vitro* antioxidant studies

Utilizing procedures described in earlier research (Dumore and Mukhopadhyay, 2020; Issac and Velumani, 2024), DPPH and ABTS experiments were carried out to assess the *in vitro* antioxidant capacity of stabilized Se-NPs. Stock solutions containing 50 µM ascorbic acid and 1 mg/mL of Se-NPs were prepared, from which working solutions of 5, 10, 15, 20, and 25 μg/mL were derived. These concentrations were fixed based on previously reported studies (Kora, 2018). The entire investigation was conducted using the mentioned doses.

#### 2.4.1 DPPH assay

In a 96-well ELISA plate, 50 µM ascorbic acid (CAS: 50-81-7; SRL) and varying doses of stabilized Se-NPs (5, 10, 15, 20, and 25 μg/mL) were added to 300 µM DPPH solution (CAS: 1898-66-4; SRL). The plates were then left in the dark for half an hour. A Thermo Fisher Scientific^®^ microplate reader measured the absorbance at 517 nm.

#### 2.4.2 ABTS assay

A reaction mixture containing 7 mM ABTS salt (CAS: 30931-67-0; SRL) and 2.45 mM potassium persulfate (1:1) was left to stand at room temperature in the dark for a day. The ABTS solution was diluted with 20X PBS to achieve an absorbance of 0.7 ± 2 at 734 nm. As a positive control, 50 µM ascorbic acid was employed. Stabilized Se-NPs at concentrations of 5, 10, 15, 20, and 25 μg/mL were used. The prepared mixtures were added to a 96-well ELISA plate containing ABTS solution and incubated for 60 min in the dark at room temperature. Using a Thermo Fisher Scientific^®^ microplate reader, the samples were measured at 734 nm.

### 2.5 *In vivo* developmental toxicity studies

#### 2.5.1 Zebrafish maintenance and embryo collection

Adult male and female zebrafish were obtained from Tarun Fish Farm in Manimangalam (latitude N 12° 55′1″ and longitude E 80° 2′29″), Chennai. Following Institutional Ethical Committee Guidelines (SU/CLAR/RD/001/2023), adult zebrafish were housed in a 19-L glass tank at 28.5°C with a 14-/10-h light/dark cycle and fed live *Artemia salina* (brine shrimp) three times daily. Breeding was initiated after 20 days of acclimatization, with two breeding groups placed in a spawning tank with a ratio of 1:1 (male: female). To prevent the female fish from swallowing the eggs, a mesh was placed at the bottom of the spawning tank. Embryos were collected 30 min after the onset of light, rinsed with freshly prepared E3 medium (Williams and Renquist, 2016), and kept at 26°C ± 1°C until the experiment (OECD, 2013; OECD, 2019).

#### 2.5.2 *In vivo* developmental toxicity test

Four hours post-fertilization (hpf), zebrafish embryos were placed in a 12-well plate, with 10 embryos (n = 30/group) per well. The control group embryos were left untreated, while the stress group embryos were exposed to CuSO_4_ (20 µM) (Nguyen et al., 2020; Herrera et al., 2021; Gao et al., 2023; Zhang P. et al., 2023), and the treatment group embryos were exposed to CuSO_4_ and treated with five different concentrations of stabilized Se-NPs (5–25 μg/mL) every 24 h. These concentrations were fixed based on previously reported studies (Kalishwaralal et al., 2015; 2016; Gao et al.,2023) and were consistently utilized throughout the study. The experiment was conducted in triplicate. Zebrafish embryo development was monitored under a microscope with 4X magnification (Issac et al., 2021; Wang et al., 2021).

### 2.6 Estimation of ROS levels in zebrafish larvae

Tricaine was used to anesthetize the larvae in each group (n = 6 per group), including the control, stress, and stabilized Se-NP treatment groups. The larvae were washed using E3 medium stained with DCFDA (20 μg/mL) dye and incubated in the dark for 1 h at room temperature. The stained larvae were washed, mounted on the clean glass, and observed visually using a fluorescent microscope (CKX53 Microscope, Japan). ImageJ software was utilized for analysis with parameters of image type: 8 bit and dark background, and the region of interest was measured with the rectangle tool (Sudhakaran et al., 2023).

### 2.7 Estimation of LPO levels in zebrafish larvae

Tricaine was used to anesthetize the larvae in each group (n = 6 per group), including the control, stress, and stabilized Se-NP treatment groups. The larvae were washed using the E3 medium and stained with the DPPP (25 μg/mL) dye and incubated at room temperature for 30 min. The stained larvae were washed, mounted on the clean glass, and visualized under a fluorescent microscope (CKX53 Microscope, Japan). ImageJ software was used for analysis with parameters of image type: 8 bit and dark background, and the region of interest was measured with the rectangle tool (Sudhakaran et al., 2023).

### 2.8 *In vivo* antioxidant studies

For enzymatic analysis, CuSO_4_-exposed larvae (n = 20/group) and adult zebrafish (n = 2/group) treated with stabilized Se-NPs (5–25 μg/mL) were homogenized in a solution containing 100 mM Tris–HCl buffer (pH 7.8 at 4°C) with 150 mM potassium chloride and 1 mM EDTA at 96 hpf. After centrifugation for 15 min at 10,000 rpm, the supernatant was utilized for further enzymatic examination (Lite et al., 2022). Protein estimation was performed using Bradford’s technique (Bradford, 1976). All experiments were conducted in triplicate.

#### 2.8.1 Superoxide dismutase (SOD) assay

An amount of 50 µL of the homogenate was mixed with a reaction mixture containing 50 mM phosphate buffer (pH 7.8), 100 µM EDTA, 750 μM NBT, 130 mM methionine, and 20 µM riboflavin. The mixture was exposed to light for 20 min, and the absorbance was measured at 560 nm (Issac et al., 2021).

#### 2.8.2 Catalase (CAT) assay

An amount of 50 µL of the sample was mixed with 100 µL of buffered H_2_O_2_ to measure catalase activity. Absorbance was recorded at 240 nm for using spectrophotometry for 2 min with 15-second intervals. This assay was conducted based on a previously published study (Velayutham et al., 2021).

#### 2.8.3 Lipid peroxidation (LPO) assay

MDA levels were measured using the thiobarbituric acid method (Velayutham et al., 2021). An amount of 100 µL of homogenized larvae and adult zebrafish sample was treated with 0.1 mL of 5% trichloroacetic acid and was incubated on ice for 15 min. Then, 0.2 mL of 0.67% thiobarbituric acid was added and incubated at 100°C in a water bath for 30 min. After chilling on ice for 20 min, the sample was centrifuged at 2,000 rpm (4°C) for 10 min, and the absorbance was measured at 535 nm (Issac et al., 2024).

#### 2.8.4 Nitric oxide (NO) assay

NO levels were measured using the Griess technique with slight modifications (Schulz et al., 1999). An amount of 100 µL of homogenized larval and adult zebrafish sample was mixed with 100 µL of Griess reagent and left at room temperature for 25 min. The absorbance was read at 540 nm.

#### 2.8.5 Reduced glutathione (GSH) and glutathione S-transferase (GST) assay

GSH and GST levels were assessed following a previously described method (Issac et al., 2021), with slight adjustments. An amount of 100 μL larval and adult zebrafish sample was combined with 50 μL of 20 mM DTNB and 150 μL of 100 mM potassium phosphate buffer at pH 7.4, and the absorbance was measured at 412 nm. For GST evaluation, 100 μL of the reaction mixture containing 10 μM GSH and 60 μM 1-chloro 2,4-dinitrobenzene was mixed with 50 μL of the larval and adult zebrafish sample, and the absorbance was noted at 340 nm.

#### 2.8.6 Estimation of acetylcholinesterase (AChE)

After exposure to CuSO_4_ and treatment with stabilized Se-NPs, samples were analyzed for AChE levels. DTNB (3.3 mM) was added to homogenized samples and incubated for 20 min, followed by the addition of acetylcholine iodide. The absorbance was measured at 412 nm at 1-minute intervals (Li et al., 2018; Issac and Velumani, 2024).

### 2.9 Cognitive behavior analysis

The locomotor abnormalities of zebrafish larvae were assessed by examining their swimming behavior pattern (Krishnan and Kang, 2019). Each exposure group (n = 3 larvae per well) was allowed to acclimatize for 10 min in a white chambered ice tray (2.5 × 3.5 cm) with 2 mL of the E3 medium without methylene blue 7 days post-fertilization. The movement of the larvae was recorded by using a commercial smartphone for 60-second at 60 frames per second in a noise-free environment at the beginning of the light cycle. Locomotion was plotted using UMATracker software (Yamanaka and Takeuchi, 2018).

### 2.10 Establishment of liver fibrosis in adult zebrafish

Healthy adult zebrafish were selected, divided into groups (n = 10/group), and placed in individual tanks—control group, CuSO_4_ group (20 μM), and CuSO_4_ + Se-NPs group (25 μg/mL)—following a 21-day exposure period. The 21-day exposure period was selected to ensure the development of chronic hepatic damage and fibrosis induced by CuSO_4_ exposure. This exposure duration was chosen in relevance to the previously reported studies (Li et al., 2023; Zhang J. et al., 2023). Following the Institutional Ethical Committee Guidelines (SU/CLAR/RD/001/2023), the fish were housed in a 19-L glass tank at 28.5°C with a 14-/10-hour light/dark cycle. They were fed live *Artemia salina* (brine shrimp) three times a day and had half of their water replaced with fresh dechlorinated water every 2 days. Three replicates were conducted for each treatment. Antioxidant enzymatic levels in adult zebrafish were evaluated through enzymatic analysis as per the protocol mentioned earlier.

### 2.11 Histological staining

Adult zebrafish (n = 2/group/week) were euthanized using tricaine and then immersed in fixative (4% paraformaldehyde) at room temperature for 24 to 48 h. The fixed zebrafish were dehydrated in ethanol, transferred to xylene for 1 h to remove excess ethanol, and then immersed in melted paraffin wax at 58°C–60°C overnight. The paraffin-infiltrated tissues were then transferred to molds to solidify. The prepared paraffin blocks were sectioned using a microtome, and the wax sections were flattened on glass slides by floating them on warm water. The hematoxylin and eosin (H&E) staining method was used, where tissue sections were stained in hematoxylin for 5–10 min, rinsed in water, washed in acid alcohol, stained in eosin for 2 min, dehydrated in ethanol, and cleared with xylene. The slides were then observed under a bright-field microscope (CKX53 Microscope, Japan) (Arman, 2021).

### 2.12 Antioxidant gene expression by real-time polymerase chain reaction (RT-PCR)

RNA was isolated from the homogenates of adult zebrafish (n = 2/group) and larvae (n = 20/group) using RDP Trio™ Reagent (SKU: MB566; HiMedia). Primers for fibrotic genes, DNA damage, and antioxidant enzymes were designed using NCBI’s Primer-BLAST (see Table 1). Gene expression was analyzed with the help of the 2x One-Step RT-PCR Master Mix (ABT018; AURA Biotech). The reverse transcription process started with a single cycle at a temperature ranging between 44°C and 50°C lasting for 15 min, followed by gene activation at 95°C for 3 min. Denaturation was repeated for 40 cycles at 95°C for 10 s. Annealing was carried out at 60°C for 45 s, and the extension process was carried out at 72°C for 15 s. The housekeeping gene β-actin is a reference gene used for normalization as it is stably expressed in most cell types. Normalization against β-actin reduces the variability in the expression levels of target genes (Santhi et al., 2025). The 2^−ΔΔCT^ method was utilized to calculate the fold change (Issac and Velumani, 2024). The analysis was conducted using Applied Biosystems StepOnePlus™.

**TABLE 1:** Primer sequences used in RT-PCR.

Gene	Forward primer (5′ to 3′)	Reverse primer (5′ to 3′)
SOD	GGT​CCG​CAC​TTC​AAC​CCT​CA	TAC​CCA​GGT​CTC​CGA​CGT​GT
CAT	AAC​TGT​GGA​AGG​AGG​GTC​GC	CGC​TCT​CGG​TCA​AAA​TGG​GC
GSR	GAT​GGG​CAC​CAT​AGC​TAA​CCC	CAT​GAG​CAG​GAA​GCA​ACA​CCC
GPx	AAC​TAC​ACT​CAG​CTT​GCG​GC	TCC​GCT​TCA​CTT​CCA​GGC​TC
Col1A1	TTG​CTT​AGA​CCT​GCG​CTT​CA	CCA​GGG​GGA​TTT​TAC​ACG​CT
DCN	CAA​TAG​CAT​CAC​CGT​TGT​GG	CCG​GAC​AGG​GTT​GCT​ATA​AA
Mt-nd1	AGC​CTA​CGC​CGT​ACC​AGT​ATT	GTT​TCA​CGC​CAT​CAG​CTA​CTG
PolG1	GAG​AGC​GTC​TAT​AAG​GAG​TAC	GAG​CTC​ATC​AGA​AAC​AGG​ACT
β-actin	AAG​CTG​TGA​CCC​ACC​TCA​CG	GGC​TTT​GCA​CAT​ACC​GGA​GC

### 2.13 Statistical analysis

All experiments in this study were conducted in triplicate and are represented as mean ± standard deviation (SD). The data were analyzed using one-way analysis of variance (ANOVA) and Dunnett’s multiple comparison test in GraphPad Prism 5.0 (GraphPad Software, Inc., San Diego, CA) (Qamar et al., 2020). The data were denoted by the symbol “*” and were considered significant with p < 0.05.

## 3 Results

### 3.1 Synthesis and characterization of selenium extracted from mussels

**FIGURE 1:**
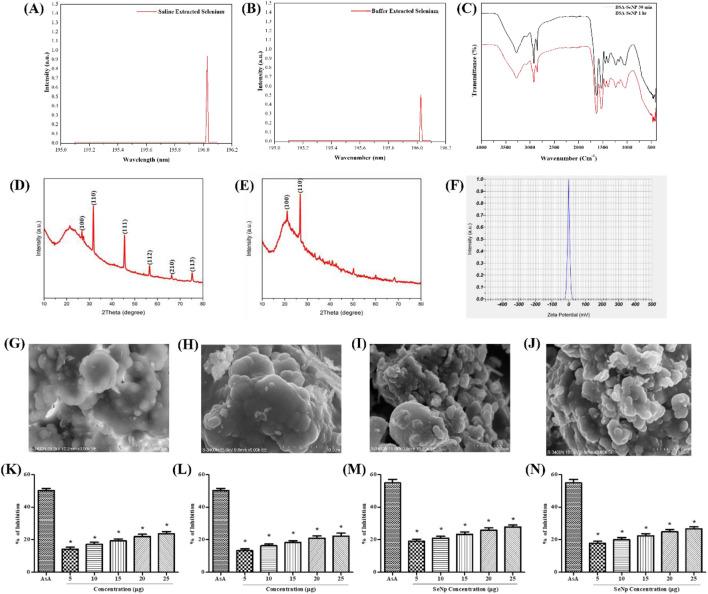
Synthesis and characterization of Se and Se-NPs: x-ray diffraction analysis of mussel-extracted selenium: **(A)** saline extraction; **(B)** buffer extraction; FTIR spectra of **(C)** stabilized Se-NPs reduced for 30 min and 1 h with BSA; ICP-OES of selenium extracted from mussel concentration of **(D)** saline extracted selenium; **(E)** buffer extracted selenium. Zeta potential of **(F)** stabilized Se-NPs reduced for 30 min with BSA. Scanning electron microscope imaging at 5x10^-3^ magnification: **(G)** non-stabilized Se-NPs reduced for 30 min; **(H)** non-stabilized Se-NPs reduced for 1 h; **(I)** stabilized Se-NPs reduced for 30 min; **(J)** stabilized Se-NPs reduced for 1 h with BSA. *In vitro* antioxidant activity: DPPH assay of **(K)** stabilized Se-NPs reduced with BSA for 30 min; **(L)** stabilized Se-NPs reduced with BSA for 1 h. ABTS assay of **(M)** stabilized Se-NPs reduced with BSA for 30 min; **(N)** stabilized Se-NPs reduced with BSA for 1 h. The data were considered significant (p < 0.05) and marked by the symbol “*.”

Saline (0.8%) and Tris–HCl buffer extraction methods were used to determine which is preferable for extracting selenium from mussels. ICP-OES results showed a distinct peak at 196.026 nm with concentrations of 0.927 mg/L for the saline extraction method (Figure 1A) and 0.509 mg/L for the buffer extraction method (Figure 1B). XRD results for selenium extracted using the saline method showed peaks at angles of 26.5076°, 31.5757°, 45.3321°, 56.3082°, 66.1258°, and 75.1616°, which correspond to the planes (100), (110), (111), (112), (210), and (113), respectively (Figure 1D). In contrast, the buffer method of extracting selenium revealed peaks at 22.1635° and 26.7682°, corresponding to planes (100) and (110), respectively (Figure 1E).

### 3.2 Synthesis and characterization of Se-NPs extracted from mussels

Se-NPs were synthesized using BSA as a reducing agent for two different periods (30 min and 1 h) and were stabilized to control aggregation. The chemical properties of the stabilized Se-NPs were detected using FTIR. The smooth and sharp peaks observed were at 3,277.830–3,274.074 cm^−1^ (medium, sharp C–H stretching alkene), 2,919.169–2,851.293 cm^−1^ (medium, sharp C–H stretching alkane), 1,628.229–1,628.660 cm^−1^ (medium, C=C stretching di-substituted alkene), 1,530.819–1,530.101 cm^-1^ (strong, N–O stretching nitro-compound), 1,454.547–1,454.339 cm^−1^ (strong, S=O stretching sulfonyl chloride), 1,396.690–1,234.989 cm^−1^ (strong, C–O stretching alkyl aryl ether), 1,042.220–1,039.471 cm^−1^ (strong, S=O stretching sulfoxide), and 471.609–409.849 cm^−1^ (strong, metal–ligand stretching) for stabilized Se-NPs reduced for both 30 min and 1 h with BSA (Figure 1C). The obtained peaks indicated the presence of metal and the reducing agent used. This also confirmed that the reduction process and duration did not alter the functional groups of the nanoparticles. Zeta potential was measured at 0.0 mV, indicating that the particles which tend to aggregate have a very low surface charge or are nearly neutral. The sharp peak implies a uniform distribution of zeta potential values (Figure 1F).

The synthesized Se-NPs showed spherical morphology. Lesser aggregation was observed in stabilized nanoparticles, compared to non-stabilized Se-NPs. The size of the non-stabilized Se-NPs was observed to be 39 and 26 nm when reduced for 30 min and 1 h, respectively (Figures 1G,H). In contrast, the size of the stabilized Se-NPs was observed to be 19 and 16 nm, showing that stabilization altered the size of the nanoparticle (Figures 1I,J). Based on the variation in size and aggregate reduction, BSA played a crucial role in enhancing the size of the nanoparticles.

### 3.3 *In vitro* antioxidant analysis of stabilized Se-NPs

#### 3.3.1 DPPH scavenging assay

The scavenging activity of ROS was assessed using the DPPH scavenging test. Ascorbic acid (AsA) showed 50% scavenging activity as a positive control. The DPPH scavenging activity of stabilized Se-NPs reduced with BSA for 30 min and 1 h was concentration-dependent. At a dose of 25 μg/mL, the highest scavenging activity was observed with 23.60% and 22.28% inhibition (Figures 1K,L).

#### 3.3.2 ABTS scavenging assay

The ROS scavenging activity was measured using the ABTS scavenging test. As a positive control, AsA exhibited 55% scavenging activity. The scavenging activity of all stabilized Se-NPs reduced with BSA for 30 min and 1 h was concentration-dependent. At a concentration of 25 μg/mL, the maximum activity was recorded with percentages of 27.79% and 24.88% (Figures 1M,N). Therefore, stabilized Se-NPs proved to be effective ROS scavengers.

### 3.4 Developmental toxicity assessment

**FIGURE 2:**
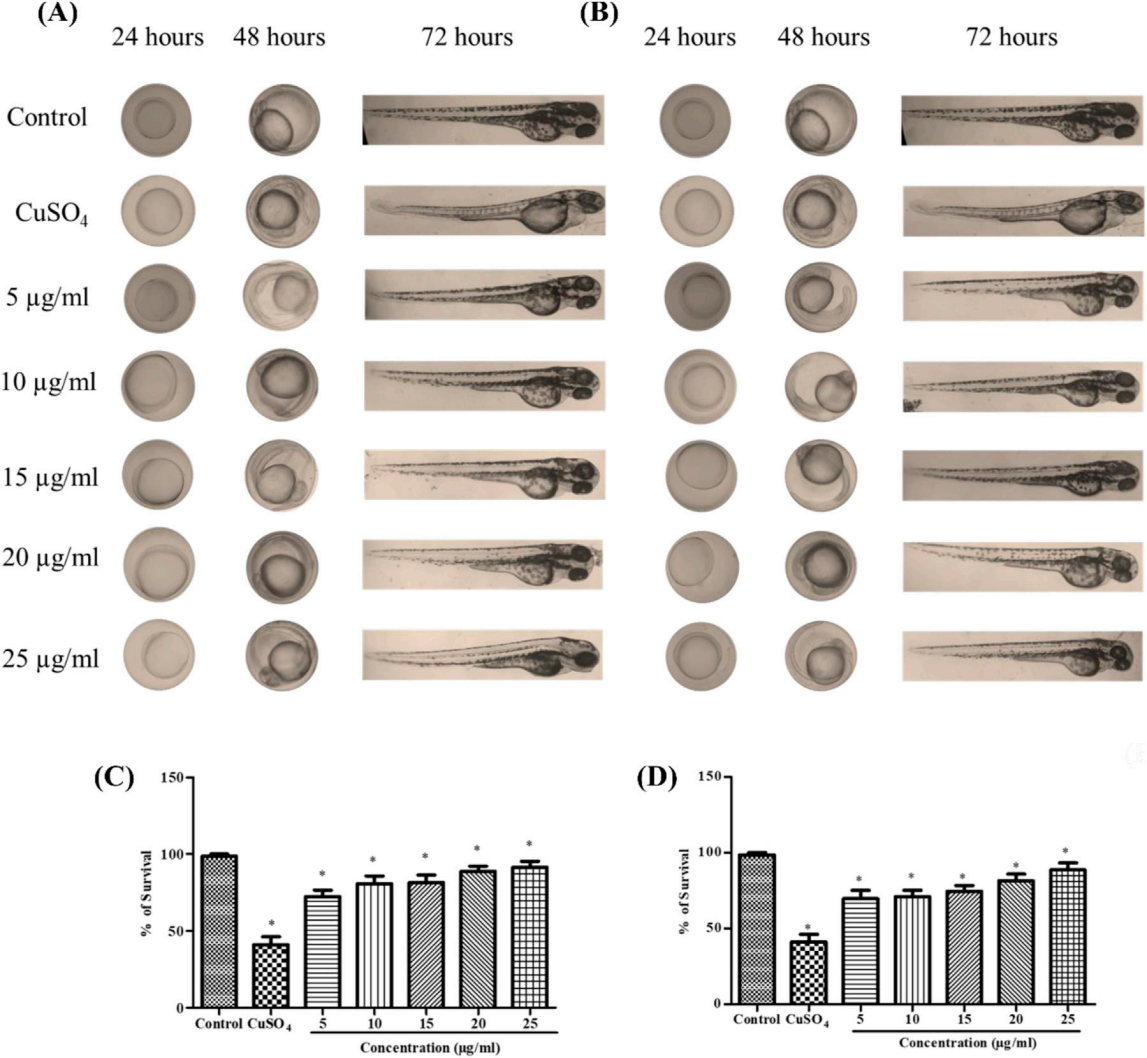
*In vivo* developmental toxicity analysis in zebrafish embryos and larvae representing the control group, stress group (CuSO_4_-induced stress), and stabilized Se-NP (5–25 μg/mL) treatment group. **(A)** Stabilized Se-NPs reduced with BSA for 30 min; **(B)** stabilized Se-NPs reduced with BSA for 1 h. Percentage of survival: **(C)** stabilized Se-NPs reduced with BSA for 30 min; **(D)** stabilized Se-NPs reduced with BSA for 1 h. The data were considered significant (p < 0.05) and marked by the symbol “*.”

The developmental toxicity assessment of Se-NPs was conducted using zebrafish embryos. In the control group, no abnormal morphological changes were observed. Groups treated with stabilized Se-NPs reduced with BSA for 30 min and 1 h (5–25 μg/mL) for 0–72 hpf showed no malformation compared to the CuSO_4_-exposed group, which exhibited malformations such as yolk-sac edema (YSE) and pericardial edema (PCE) (Figures 2A,B). In comparison to the CuSO_4_-exposed group, stabilized Se-NPs reduced with BSA for 30 min and 1 h showed a lower mortality rate at 96 hpf (Figures 2C,D).

### 3.5 Localization of cellular ROS

**FIGURE 3:**
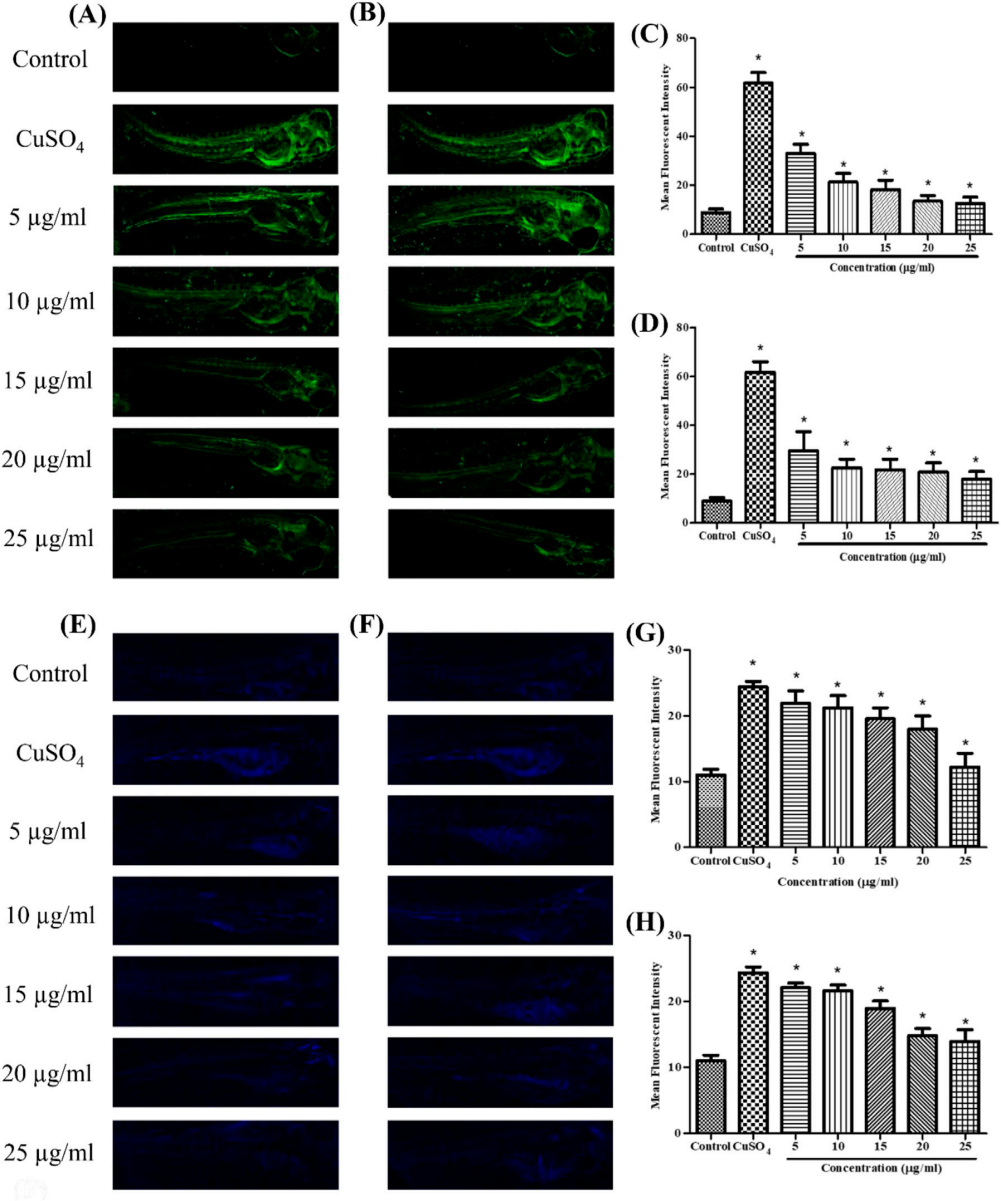
Live cell imaging: *in vivo* DCFDA and DPPP staining in the zebrafish larvae model at 96 hpf. DCFDA staining: **(A)** stabilized Se-NPs reduced with BSA for 30 min; **(B)** stabilized Se-NPs reduced with BSA for 1 h. Mean fluorescent intensity: **(C)** stabilized Se-NPs reduced with BSA for 30 min; **(D)** stabilized Se-NPs reduced with BSA for 1 h. DPPP staining: **(E)** stabilized Se-NPs reduced with BSA for 30 min; **(F)** stabilized Se-NPs reduced with BSA for 1 h. Mean fluorescent intensity: **(G)** stabilized Se-NPs reduced with BSA for 30 min; **(H)** stabilized Se-NPs reduced with BSA for 1 h. The data were considered significant (p < 0.05) and marked by the symbol “*.”

Using DCFDA fluorescent labeling, zebrafish larvae (96 hpf) were assessed for intracellular ROS. The mean fluorescence intensity (MFI) for the control group was 9.23. ROS levels in zebrafish larvae exposed to CuSO_4_ were 60.72. In CuSO_4_-induced zebrafish larvae, treatment with stabilized Se-NPs significantly (p < 0.05) decreased cellular ROS levels compared to that in the untreated stress group (Figures 3A,B; Supplementary Figure S1). Lower ROS levels were observed at concentrations of 12.60 and 17.97 MFI in the 25 μg/mL stabilized Se-NPs treated with BSA for 30 min and 1 h (Figures 3C,D).

### 3.6 Determination of live cell lipid peroxidation

Using DPPP fluorescent staining, zebrafish larvae (96 hpf) were observed for LPO. The control group showed 11.02 MFI. ROS levels in zebrafish larvae exposed to CuSO_4_ were 24.35 MFI. In CuSO_4_-exposed zebrafish larvae, treatment with stabilized Se-NPs significantly decreased the LPO compared to that in the untreated stress group (Figures 3E,F; Supplementary Figure S2). Stabilized Se-NPs at the 25 μg/mL concentration reduced the LPO to 11.35 and 13.17 MFI (Figures 3G,H).

### 3.7 *In vivo* enzymatic assay of stabilized Se-NPs

#### 3.7.1 SOD assay

**FIGURE 4:**
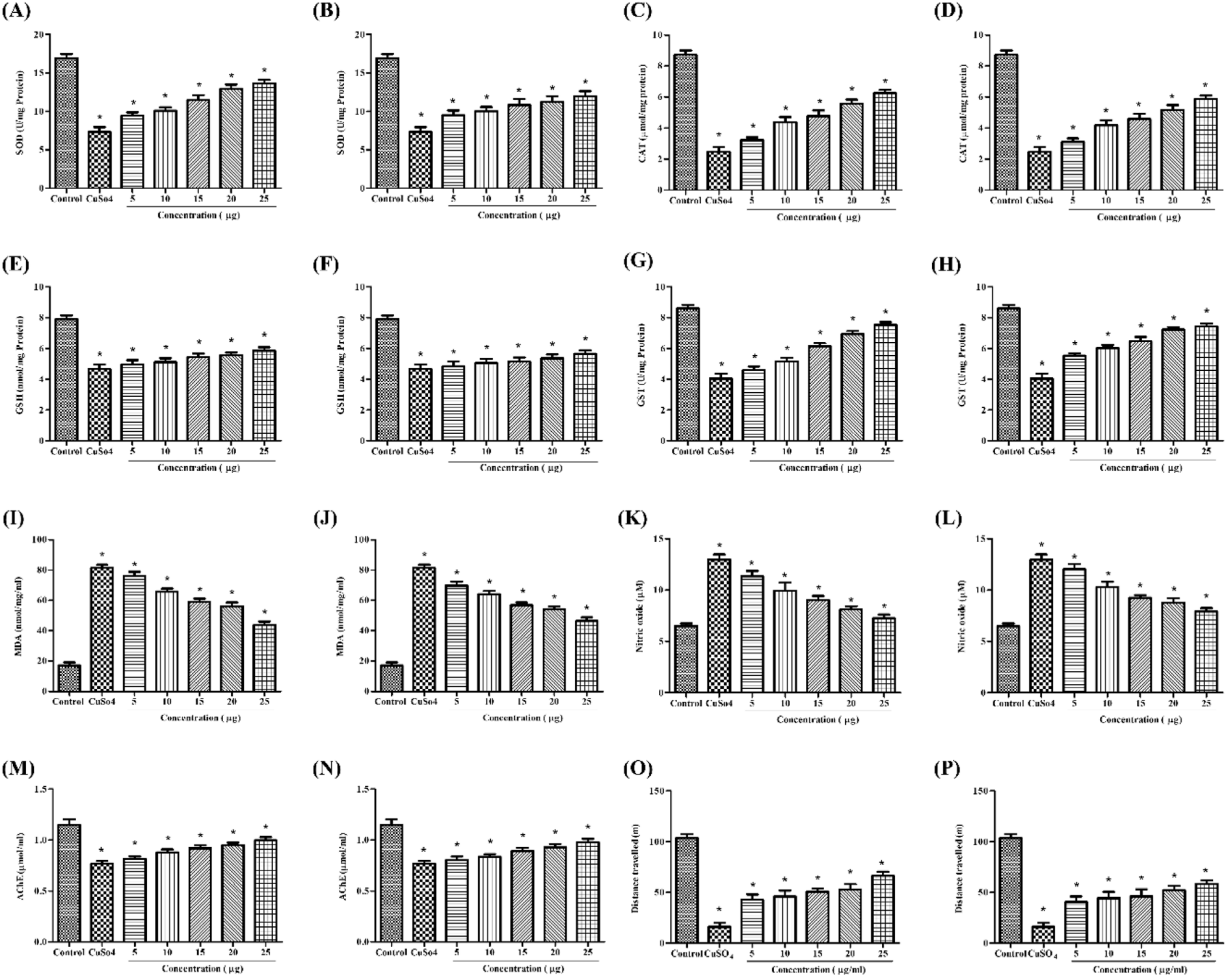
*In vivo* antioxidant activity: SOD assay of **(A)** stabilized Se-NPs reduced with BSA for 30 min and **(B)** stabilized Se-NPs reduced with BSA for 1 h. CAT assay of **(C)** stabilized Se-NPs reduced with BSA for 30 min and **(D)** stabilized Se-NPs reduced with BSA for 1 h. GSH assay of **(E)** stabilized Se-NPs reduced with BSA for 30 min and **(F)** stabilized Se-NPs reduced with BSA for 1 h. GST assay of **(G)** stabilized Se-NPs reduced with BSA for 30 min and **(H)** stabilized Se-NPs reduced with BSA for 1 h. LPO assay of **(I)** stabilized Se-NPs reduced with BSA for 30 minand **(J)** stabilized Se-NPs reduced with BSA for 1 h. NO assay of **(K)** stabilized Se-NPs reduced with BSA for 30 min and **(L)** stabilized Se-NPs reduced with BSA for 1 h. AChE assay of **(M)** stabilized Se-NPs reduced with BSA for 30 min and **(N)** stabilized Se-NPs reduced with BSA for 1 h. Locomotory behavior of **(O)** stabilized Se-NPs reduced with BSA for 30 min and **(P)** stabilized Se-NPs reduced with BSA for 1 h. The data were considered significant (p < 0.05) and marked by the symbol “*”.

A homogenized sample of zebrafish larvae treated with stabilized Se-NPs after exposure to CuSO_4_ was used to measure SOD levels. Comparing the CuSO_4_-exposed group to the control group (16.90 U/mg of protein), the SOD levels were observed to drop significantly to 7.33 U/mg of protein (a 57% reduction). The concentration-dependent rise in enzymatic activity was demonstrated by 25 μg/mL stabilized Se-NPs, which was reduced with BSA for 30 min and 1 h, restoring the enzymatic levels to 13.65 U/mg of protein and 11.97 U/mg of protein, respectively (Figures 4A,B).

#### 3.7.2 CAT assay

A homogenized sample of zebrafish larvae treated with stabilized Se-NPs after exposure to CuSO_4_ was used to measure CAT levels. CAT levels were significantly lower in the CuSO_4_-exposed group (2.49 μmol/mg of protein) than in the control group (8.72 μmol/mg of protein). CuSO_4_-exposed zebrafish larvae treated with stabilized Se-NPs reduced with BSA for 30 min and 1 h at the concentration of 25 μg/mL showed concentration-dependent increases in total CAT levels of 6.25 μmol/mg and 5.86 μmol/mg, respectively (Figures 4C,D).

#### 3.7.3 Estimation of GSH activity

GSH activity was significantly lower in the CuSO_4_-exposed group (4.67 nmol/mg protein) than in the control group (7.89 nmol/mg protein). In contrast, CuSO_4_-exposed zebrafish larvae treated with 25 μg/mL stabilized Se-NPs reduced with BSA for 30 min and 1 h greatly improved the GSH activity. As seen in Figures 4E,F, the GSH levels retained were 5.85 and 5.64 nmol/mg of protein, respectively.

#### 3.7.4 Estimation of the GST activity

In comparison to the control group (8.59 U/mg protein), the CuSO_4_-exposed group exhibited a substantial decrease in GST activity (4.04 U/mg protein). After treatment with stabilized Se-NPs reduced with BSA (25 μg/mL) for 30 min and 1 h, GST activity increased markedly to 7.52 and 7.41 U/mg of protein, respectively (Figures 4G,H).

#### 3.7.5 Lipid peroxidation assay

MDA levels were significantly higher in the CuSO_4_-exposed stress group (81.69 nmol/mg/mL) than in the control group (17.09 nmol/mg/mL). In zebrafish larvae exposed to CuSO_4_, stabilized Se-NPs reduced with BSA for 30 min and 1 h at a dose of 25 μg/mL considerably decreased the MDA levels by 43.77 and 46.49 nmol/mg/mL, respectively (Figures 4I,J).

#### 3.7.6 Estimation of NO levels

The Griess reagent assay was used to measure NO levels. Zebrafish larvae exposed to CuSO_4_ exhibited higher NO levels (12.9 µM) than those in the control group (6.49 µM). NO levels decreased dose-dependently, and a significant decline was observed with 25 μg/mL stabilized Se-NPs reduced with BSA for 30 min and 1 h, showing values of 7.27 µM and 7.93 µM, respectively (Figures 4K,L).

#### 3.7.7 Estimation of AChE activity

Zebrafish larvae exposed to CuSO_4_ demonstrated a considerable drop in AChE levels, from 1.14 μmol/mL to 0.76 μmol/mL, compared to the control group. When CuSO_4_-exposed zebrafish larvae were treated with 25 μg/mL stable Se-NPs decreased with BSA for 30 min and 1 h, respectively, AChE levels increased in a dose-dependent manner to reach 1.04 and 0.97 μmol/mL, respectively (Figures 4M,N).

### 3.8 Estimation of locomotor activity

The neurobehavior of zebrafish larvae was assessed by measuring the distance they traveled (in meters), allowing us to determine their locomotor activity and cognitive changes in the central nervous system. Zebrafish larvae exposed to CuSO_4_ traveled 16.2 m, likely due to stress effects. In the CuSO_4_-exposed zebrafish larval group, treatment with 25 μg/mL of stabilized Se-NPs reduced with BSA for 30 min and 1 h enhanced cognitive behavior by restoring their locomotor activity, thus covering distances up to 66.26 m and 58.57 m. The control group traveled 102.60 m, demonstrating normal cognitive behavior (Figures 4O,P).

### 3.9 Histopathology analysis

**FIGURE 5:**
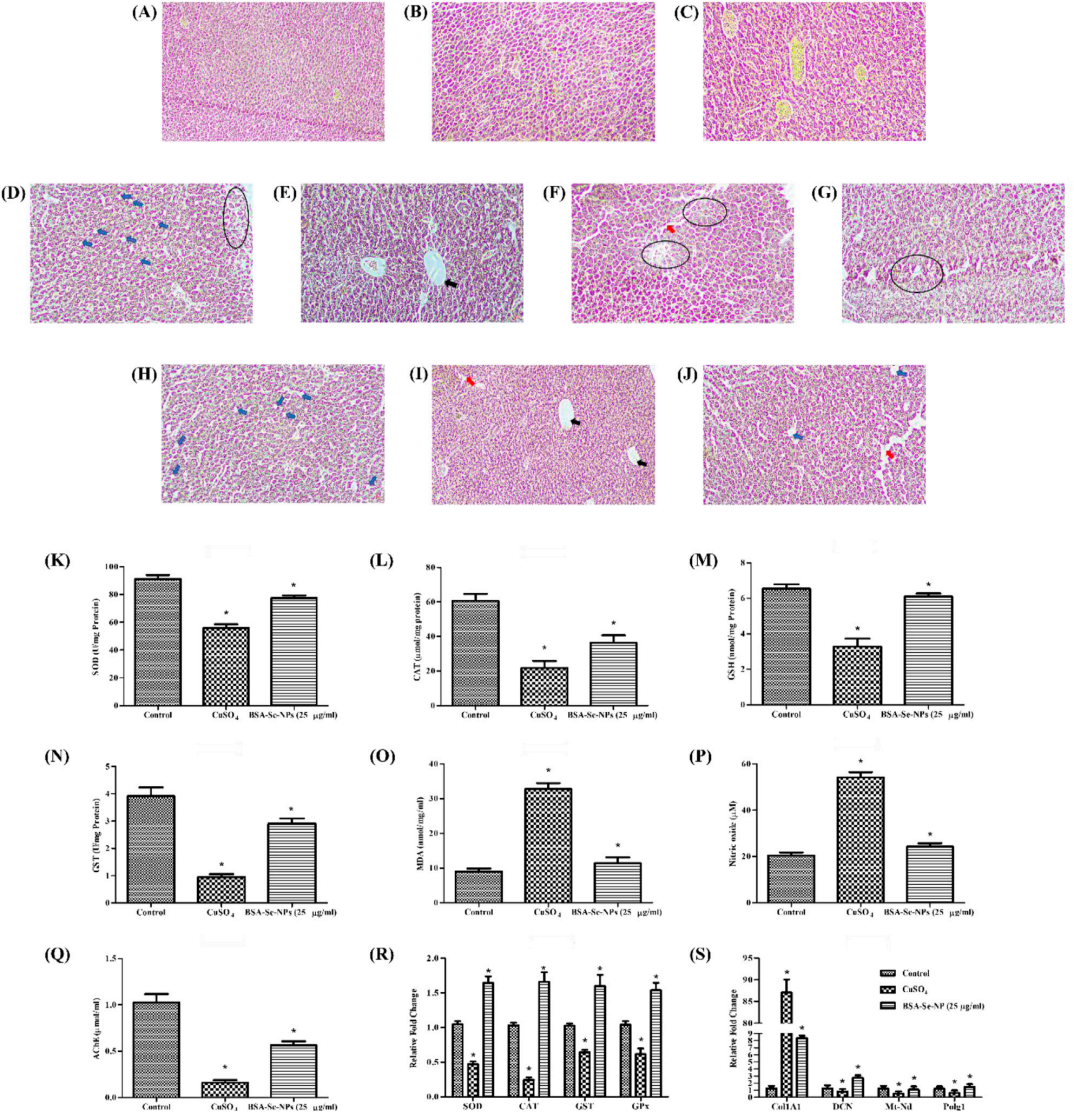
*In vivo* hepatic fibrosis model and antioxidant activity: histopathology of control (healthy) at **(A)** day 7, **(B)** day 14, and **(C)** day 21; stress group (CuSO_4_) at **(D)** day 7, **(E,F)** day 14, and **(G)** day 21; treatment group (25 μg/mL stabilized Se-NPs reduced with BSA for 30 min) at **(H)** day 7, **(I)** day 14, and **(J)** day 21. The symbols indicate the following: hepatic stellate cells (blue arrow); sinusoidal dilation (red arrow); vacuolation (black arrow), and degeneration (round), with scale bar (10 µm). Antioxidant enzymatic estimation: **(K)** SOD assay; **(L)** CAT assay; **(M)** GSH assay; **(N)** GST assay; **(O)** LPO assay; **(P)** NO assay; **(Q)** AChE assay. RT-PCR of **(R)** antioxidant genes and **(S)** hepatic fibrosis markers. The data were considered significant (p < 0.05) and marked by the symbol “*.”

The histopathology of adult zebrafish liver was analyzed to determine morphological alterations. In control zebrafish, hepatocytes and nuclei were observed (Figures 5A–C). Liver histology of zebrafish exposed to CuSO_4_ distinctly showed the formation of vacuoles on day 7, dilation in the sinusoidal area, and hepatocyte degeneration on days 14 and 21, with noticeable alterations (Figures 5D–G). However, the treatment group showed damage in liver morphology such as vacuolation on day 14 and sinusoidal dilation on day 21, but the progression was observed to be delayed (Figures 5H–J). Degeneration of hepatocytes was not observed in the treatment group.

### 3.10 Enzymatic assays

After the treatment period, adult zebrafish were homogenized to study the antioxidant enzymatic levels. The control group had 91.27 U/mg of SOD and 60.54 μmol/mg of CAT. Compared to the CuSO_4_-induced stress group with an SOD level of 55.88 U/mg and CAT level of 21.78 μmol/mg, the SOD and CAT levels were higher in the stabilized Se-NP (25 μg/mL)-treated group with 77.64 U/mg and 36.35 μmol/mg (Figures 5K,L). GSH and GST levels were significantly maintained in the stabilized Se-NP-treated group with 6.10 nmol/mg and 2.90 U/mg. In contrast, the CuSO_4_-induced stress group had 3.27 nmol/mg of GSH and 0.94 U/mg of GST. The control group had 6.53 nmol/mg of GSH and 3.91 U/mg of GST (Figures 5M,N).

MDA levels in the CuSO_4_ stress group were notably increased to 32.73 nmol/mg/mL compared to that in the control group, which was 9.01 nmol/mg/mL. Stabilized Se-NPs reduced the MDA levels to 11.35 nmol/mg/mL compared to that in the stress group (Figure 5O). The control group showed lower NO levels with 20.38 µM. In contrast, the stress group exhibited 54.13 µM, which was regulated to 24.20 µM in the treatment group that received stabilized Se-NPs (Figure 5P). The neuroprotectant AChE was significantly low in the CuSO_4_-induced stress group with 0.15 μmol/mL. Stabilized Se-NPs protected the AChE levels up to 0.56 μmol/mL compared to that in the stress group. The control group had higher AChE levels of 1.02 μmol/mL when compared to the stress group and treatment group (Figure 5Q).

### 3.11 Expression of antioxidant genes

Cellular homeostasis is disrupted by ROS generation, which is triggered by a deficiency of selenium, a crucial component of antioxidant enzymes. The larvae were subjected to CuSO_4_-induced stress, which effectively influenced the expression of antioxidant genes. CuSO_4_-exposed zebrafish larvae treated with stabilized Se-NPs reduced for 30 min and 1 h with BSA efficiently mitigated ROS, as demonstrated by the significant upregulation of SOD (1.64-fold), CAT (1.65-fold), glutathione-S-transferase (GST) (1.59-fold), and glutathione peroxidase (GPX) (1.53-fold) genes (Figure 5R). Likewise, stabilized Se-NPs also delayed hepatic damage, confirmed by the downregulation of Col1A1 (8.34-fold) and upregulation of DCN (2.75-fold), Mt-nd (1.07-fold), and PolG (1.49-fold) genes (Figure 5S).

## 4 Discussion

Selenium extraction from mussels was performed using saline and buffer extraction methods (Kristan et al., 2015). Our extraction method was cost-effective and sustainable, utilizing mussels as a natural source of selenium and reducing the need for expensive chemicals like sodium selenite. The procedure involves low-energy steps, making it feasible for large-scale applications. Se-NPs were synthesized using BSA as the reducing agent (Chandramohan et al., 2019). BSA is a stabilizing agent (Cao et al., 2019). Selenium peaks were observed at 196.03 (Tyburska et al., 2010), confirming its presence. The study showed that mussel-derived selenium exhibited peaks similar to those reported by Hassanien et al. on dye degradation, with XRD peaks at 26.5076°, 31.5757°, and 45.3321° corresponding to the (100), (110), and (111) planes for Se-NPs, demonstrating selenium’s presence (Hassanien et al., 2019). FTIR spectrum peaks at 3,277.830, 2,919.169, 1,628.229, 1,530.819, 1,454.547, 1,396.690, and 1,042.220 cm^−1^ represented functional groups, while those at 471.609 and 409.849 cm^−1^ indicated metal–ligand stretching, respectively. The presence of hydroxyl or amide groups from BSA was indicated by the peak at 3,277.830 cm^−1^, while that at 2,919.169 cm^−1^ likely came from BSA–protein backbones. The absorption peaks at 1,628.229 and 1,530.819 indicated amide bands and secondary structure changes upon the Se-NP interaction, with the peak at 1,396.690 showing the presence of ether or carboxyl groups, which were potentially involved in Se-NP stabilization, and those at 471.609 and 409.849 cm^−1^ indicating Se-NP binding with protein functional groups. In 2016, a study by Kalishwaralal et al. on a novel one-pot green synthesis of Se-NPs observed similar peaks (Kalishwaralal et al., 2016). SEM observation showed that non-stabilized Se-NPs were spherical, while stabilized Se-NPs were less aggregated than Se-NPs. A study by Chung et al. on green-synthesized BSA-coated Se-NPs reported that the nanoparticles were spherical in shape with a size ranging from 12.2 to 53.9 nm (Chung et al., 2020). Se-NPs potentially scavenged ROS, which was confirmed through cell-free DPPH and ABTS assays. A study by Wang et al. in 2024 on the biosynthesis of selenium nanoparticles by *Bacillus licheniformis* showed a similar scavenging activity (Wang et al., 2024). In 2024, a study by Vasanthakumar et al. on green synthesis, characterization, and functional validation of bio-transformed Se-NPs reported that Se-NPs exhibited 74% ROS scavenging activity at a concentration of 1 mg/mL (Vasanthakumar et al., 2024). A study on the antimicrobial and antioxidant potential of Se-NPs with *Olea ferruginea* fruit extract showed 81.12% ABTS scavenging activity (Hassan et al., 2022).

This study highlights the role of metals, such as copper, in inducing oxidative stress and organ damage, which is a key factor contributing to hepatic dysfunction and neurodegeneration via the brain–liver axis (Zhong et al., 2023; Hake et al., 2025). Metal-induced ROS generation can lead to mitochondrial dysfunction, inflammatory responses, and cellular damage, all of which are implicated in neurodegenerative disorders such as Parkinson’s and Alzheimer’s disease (Cheng et al., 2021). This preliminary research was conducted using the zebrafish model, which shares ∼70% genome similarity with humans and is a widely recognized model for studying oxidative stress, liver toxicity, and neurodegeneration (Howe et al., 2013; Adhish and Manjubala, 2023).

A previous study on embryo toxicity profile by Kalishwaralal et al. reported that at 15–25 μg/mL, malformations such as tail malformation and pericardium-sac edema were observed (Kalishwaralal et al., 2016). In our study, malformations were noted in the CuSO_4_-induced stress group, while the larvae treated with Se-NPs did not show any developmental malformations. Fluorescent staining showed a concentration-dependent decrease in ROS and LPO levels. Raju et al. reported a similar methodology to measure the ROS and LPO levels in zebrafish larvae exposed to H_2_O_2_ stress, which revealed higher intensity in the stress group than in the treatment groups (Raju et al., 2021).

Enzymatic analysis evaluated the decline and restoration of anti-oxidative enzymes, showing that CuSO_4_ exposure led to a significant impact by decreasing the first-line defense including SOD and CAT, which eventually triggered the levels of MDA and NO in both larvae and adult zebrafish. Se-NP treatment restored the SOD and CAT levels, although the larvae and adult zebrafish were exposed to CuSO_4_-induced stress. A study by Zhou et al. reported that CuSO_4_ diminished the SOD levels (Zhou et al., 2023). In 2023, Au et al. stated that Se-NPs improved the SOD levels in plasma (Li et al., 2022; Au et al., 2023). A study by Zhou et al. on CuSO_4_ exposure to oxidative stress stated that CuSO_4_ reduced the CAT levels (Zhou et al., 2023). Dawit Moges et al. stated that Se-NPs enhanced CAT levels and improved hepatic antioxidant defense mechanism (Dawit Moges et al., 2022), thus regulating the MDA and NO levels. A study by Salaramoli et al. reported that Se-NPs significantly reduced the MDA levels and improved the total antioxidant capacity (Salaramoli et al., 2023). A study by Buacheen et al. on antioxidant and anti-inflammatory activities of nano-selenium reported that Se-NPs exhibited significant anti-inflammatory activity by downregulating the NO levels (Buacheen et al., 2023). Similarly, levels of detoxifying enzymes such as GSH and GST were restored in Se-NP-treated larvae and adult zebrafish, showing the protective effects of Se-NPs against oxidative stress. In 2022, Othman et al. stated that Se-NPs notably increased GSH levels (Othman et al., 2022). Zahran et al. reported that GST levels were upregulated upon treatment with Se-NPs (Zahran et al., 2024).

To determine their effect on cerebral health, AChE levels and locomotor behavior were analyzed. The results showed that AChE levels were maintained in the group treated with Se-NPs, while the stress group had reduced levels of AChE, confirming that Se-NPs can be an effective neuroprotector. A study by Khalil et al. on Se-NPs imparting robust neuroprotection showed that Se-NPs significantly restored AChE levels and acted as a neuroprotector (Khalil et al., 2022). In 2010, a study by Pinton et al. reported that Se-NPs can retain memory and learning impairments triggered by streptozotocin (Pinton et al., 2010). The larvae that received treatment efficiently traveled farther distances than the larvae in the stress group. In 2019, a study on behavioral impairments and oxidative stress by Sarasamma et al. reported that behavioral alteration is greatly associated with the central nervous system and can be assessed based on their swimming pattern (Sarasamma et al., 2019).

The CuSO_4_-exposed zebrafish showed marked liver damage, characterized by the formation of vacuoles, sinusoidal dilation, and hepatocyte degeneration, with the damage progressively worsening over 7, 14, and 21 days. These findings suggest that CuSO_4_-induced oxidative stress severely impacts liver morphology, aligning with its known hepatotoxic effects. However, the treatment group, which received Se-NPs, significantly attenuated these damaging effects. While vacuolation and sinusoidal dilation were still present on days 14 and 21, respectively, the damage progression was notably delayed compared to that in the CuSO_4_ stress group. Most importantly, the absence of hepatocyte degeneration in the treatment group suggests that Se-NPs effectively preserved liver cell integrity. The delayed onset of liver pathology further underscores the potential of Se-NPs as a therapeutic intervention for liver injury (Arman, 2021). A study by Loeschner et al. (2014) demonstrated that Se-NPs exhibited distinct pharmacokinetic properties compared to ionic selenium, with prolonged circulation time, controlled release, and selective tissue accumulation (Loeschner et al., 2014). Similarly, Li et al. (2024) highlighted the role of nanoparticle surface chemistry in enhancing bioavailability and cellular uptake (Li et al., 2024). These findings suggest that the physicochemical properties of Se-NPs, such as size, charge, and coating, likely influence their absorption and biodistribution in zebrafish.

**FIGURE 6:**
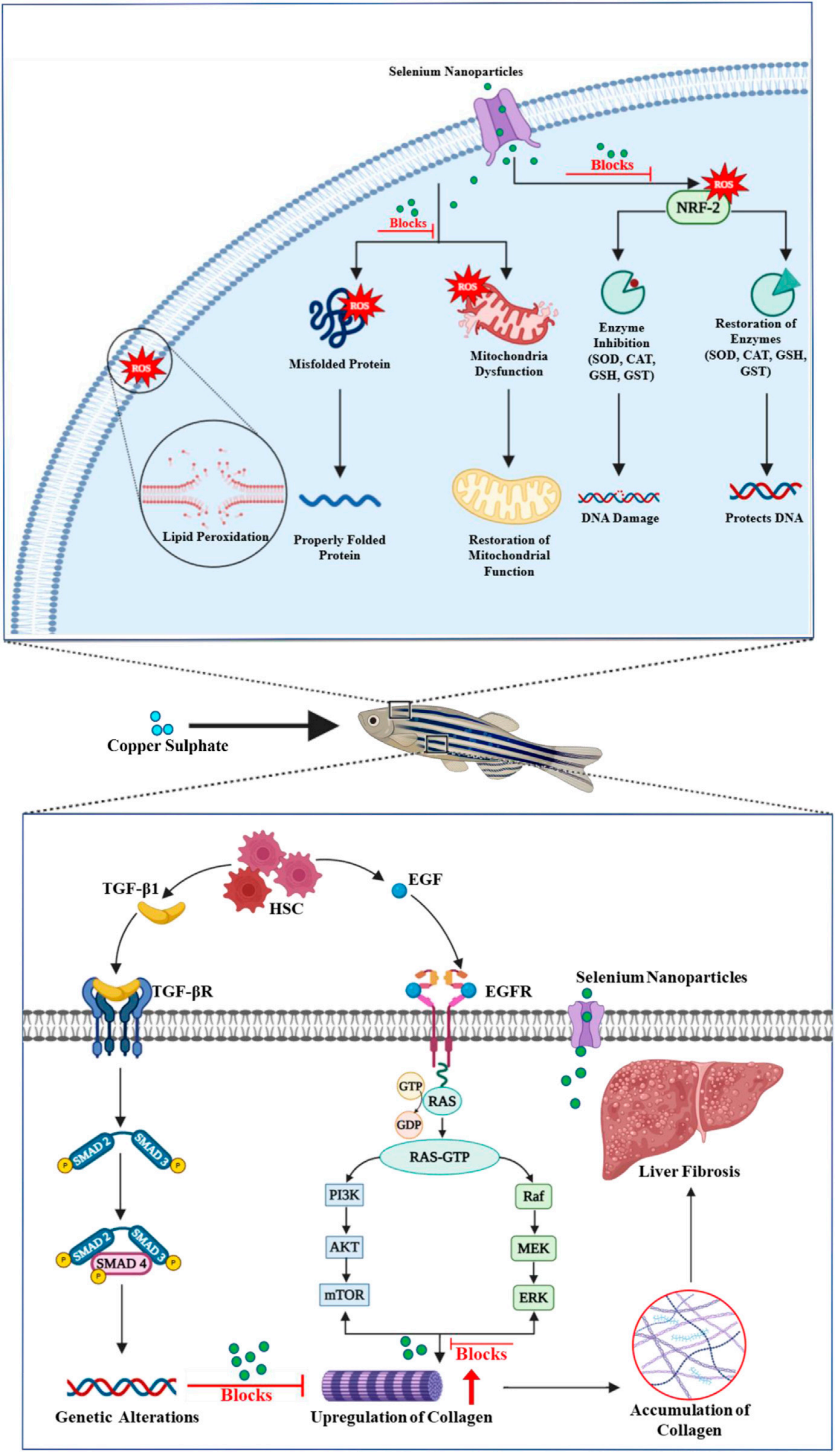
Schematic representation of the multifaceted protective role of selenium nanoparticles in preventing copper sulfate-induced liver fibrosis and maintaining cellular and mitochondrial integrity.

The study demonstrated that Se-NPs stabilized with BSA mitigated CuSO_4_-induced oxidative stress in zebrafish larvae by significantly enhancing antioxidant defenses and delaying hepatic damage. Notably, Se-NPs increased the expression of key antioxidant genes, indicating robust ROS neutralization, which helps maintain cellular homeostasis. The observed downregulation of Col1A1 suggests reduced collagen deposition, potentially slowing fibrosis, while the upregulation of DCN supports matrix remodeling, which aids in preserving liver structure (Zhu et al., 2013; Liang et al., 2021; Aljagthmi et al., 2024). Additionally, increased levels of Mt-nd and PolG suggest enhanced mitochondrial function and DNA repair, which are essential for cell recovery and resilience against oxidative stress (Valdivieso et al., 2007; Foote et al., 2018; Zhu et al., 2023). Collectively, Se-NPs showed a protective role in managing oxidative stress-induced liver damage, offering a promising strategy for liver damage prevention (Figure 6).

## 5 Conclusion

Liver fibrosis is a significant global health concern and an early stage in the progression toward cirrhosis and liver failure. CuSO_4_ is a common hepatotoxic agent that induces oxidative stress, leading to liver cell injury and fibrosis progression. Excess ROS generation in the liver accelerates fibrosis development. Extracting selenium from mussels offers an eco-friendly approach to obtaining this nutrient, which can be further processed into bioactive forms such as Se-NPs. Stabilized Se-NPs show better size reduction than non-stabilized Se-NPs. Treatment with stabilized Se-NPs effectively reduces developmental malformations and free radicals.

*In vivo* enzymatic assays showed that stabilized Se-NP treatment improved the antioxidant enzymatic levels in CuSO_4_-exposed larvae by reducing LPO and NO levels. Stabilized Se-NPs also enhanced the AChE levels and protected the cognitive behavior of larvae. The histopathological results further confirmed that Se-NP treatment delayed the progression of liver damage such as hepatic stellate cells, vacuoles, sinusoidal dilation, and hepatocyte degeneration. Gene expressions confirmed that Se-NPs effectively mitigated CuSO_4_-induced hepatic damage in zebrafish, as evidenced by the upregulation of antioxidant, DCN, Mt-nd, and PolG genes and the downregulation of the Col1A1 gene. The ability of Se-NPs to interact more efficiently with ROS, combined with their enhanced bioavailability, makes them a powerful tool in the fight against liver fibrosis and other oxidative stress-related conditions. These findings suggest that Se-NPs can regulate ROS levels and protect against oxidative stress-induced cellular and hepatic damage. Moreover, the neuroprotective potential of Se-NPs, linked through the brain–liver axis, opens promising avenues for further research on their therapeutic applications in neurodegenerative disorders.

## Data Availability

The datasets presented in this study can be found in online repositories. The names of the repository/repositories and accession number(s) can be found in the article/Supplementary Material.
