# Supplementary material for: Evaluating the therapeutic potential of BSA-reduced mussel-derived selenium nanoparticles to mitigate copper sulfate-induced hepatic damage and neurodegeneration in a zebrafish model
Source: Front Genet. 2025 May 19;16:1522370. doi: 10.3389/fgene.2025.1522370 (PMC12127314; doi:10.3389/fgene.2025.1522370)
Supplement: Supplementary file 1 [file DataSheet1.docx]

**Title: Evaluating the Therapeutic Potential of BSA-Reduced Mussel-Derived Selenium Nanoparticles to Mitigate Copper Sulfate-Induced Hepatic Damage and Neurodegeneration in Zebrafish Model**

**Author Name**: Suganiya Umapathy and Ieshita Pan*

**Address:** Institute of Biotechnology, Department of Medical Biotechnology and Integrative Physiology, Saveetha School of Engineering, Saveetha Institute of Medical and Technical Sciences, Thandalam, Chennai, 602 105, Tamil Nadu, India

***Correspondence:** [bony.iesk@gmail.com](mailto:bony.iesk@gmail.com); [ieshitapan.sse@saveetha.com](mailto:ieshitapan.sse@saveetha.com)

**Running Head: Mussel-Derived Nano-Se Mitigate Cu-Induced Hepatic Damage and Neurodegeneration in Zebrafish Model**

**Supplementary Figures**

**
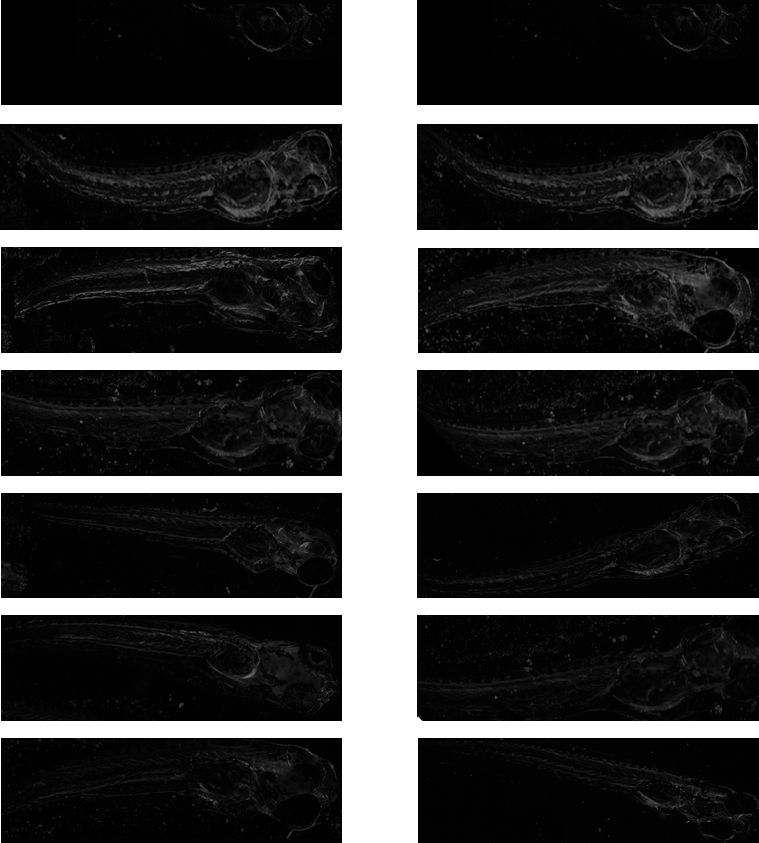
**

**Supplementary Figure 1:** DCFDA Staining: (A) stabilized Se-NPs reduced with BSA for 30 minutes; (B) stabilized Se-NPs reduced with BSA for 1 hour.

**
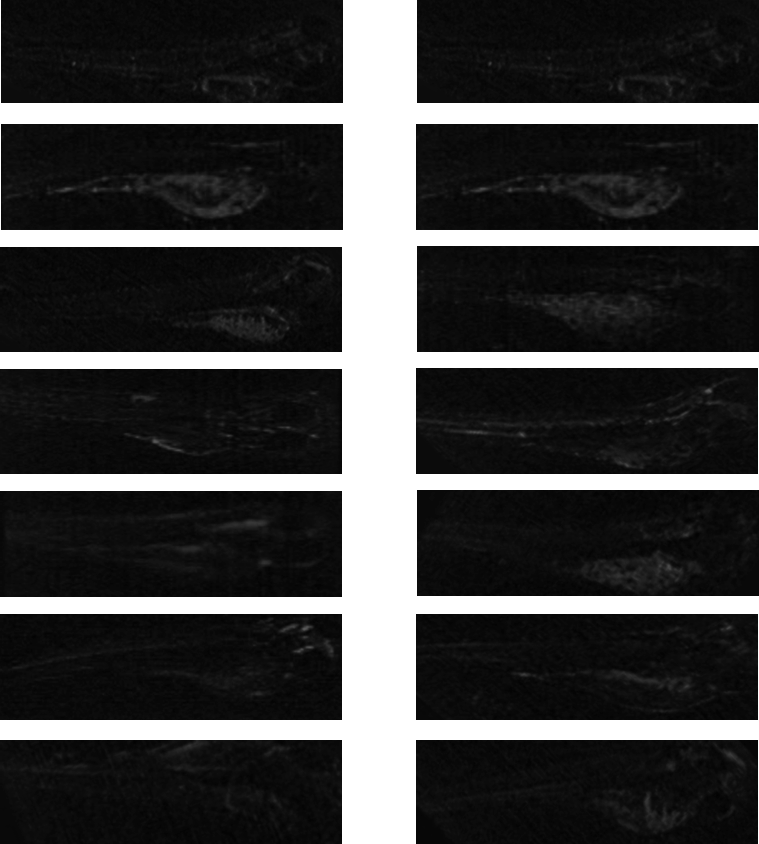
**

**Supplementary Figure 2:** DPPP Staining: (A) stabilized Se-NPs reduced with BSA for 30 minutes; (A) stabilized Se-NPs reduced with BSA for 1 hour.
